# Guidelines for Robot-to-Human Handshake From the Movement Nuances in Human-to-Human Handshake

**DOI:** 10.3389/frobt.2022.758519

**Published:** 2022-03-28

**Authors:** John-John Cabibihan, Ahmed El-Noamany, Abdelrahman Mohamed Ragab M. Ahmed, Marcelo H. Ang

**Affiliations:** ^1^ Department of Mechanical and Industrial Engineering, Qatar University, Doha, Qatar; ^2^ Department of Mechanical Engineering, National University of Singapore, Singapore, Singapore

**Keywords:** handshake, human-robot handshake, reaching, pHRI, grasping

## Abstract

The handshake is the most acceptable gesture of greeting in many cultures throughout many centuries. To date, robotic arms are not capable of fully replicating this typical human gesture. Using multiple sensors that detect contact forces and displacements, we characterized the movements that occured during handshakes. A typical human-to-human handshake took around 3.63 s (SD = 0.45 s) to perform. It can be divided into three phases: reaching (M = 0.92 s, SD = 0.45 s), contact (M = 1.96 s, SD = 0.46 s), and return (M = 0.75 s, SD = 0.12 s). The handshake was further investigated to understand its subtle movements. Using a multiphase jerk minimization model, a smooth human-to-human handshake can be modelled with fifth or fourth degree polynomials at the reaching and return phases, and a sinusoidal function with exponential decay at the contact phase. We show that the contact phase (1.96 s) can be further divided according to the following subphases: preshake (0.06 s), main shake (1.31 s), postshake (0.06 s), and a period of no movement (0.52 s) just before both hands are retracted. We compared these to the existing handshake models that were proposed for physical human-robot interaction (pHRI). From our findings in human-to-human handshakes, we proposed guidelines for a more natural handshake movement between humanoid robots and their human partners.

## 1 Introduction

The handshake is a gesture being exchanged when meeting or parting with someone (Hall and Hall, 1983; Black, 2011; Melnyk and Henaff, 2019). The handshake communicates goodwill, appreciation, empathy, and gratitude. A good handshake may convey an individual’s sociability, competence, and trustworthiness while a poor handshake may communicate introversion and shyness (Astrom and Thorell, 1996; Chaplin et al., 2000; Bernieri and Petty, 2011). It was shown that a handshake had a positive influence on the way an individual evaluated the interaction partner and that the handshake promoted an interest for further social interaction (Dolcos et al., 2012; Schroeder et al., 2019). The handshake requires physical contact at a distance which is not generally considered to be intrusive to the personal space of another. Physical touch is a biological need and it is associated with warmth and closeness (Davis, 1999; Cabibihan et al., 2015).

The handshake gesture is now being passed on to humanoid robots for the same reasons why humans exchange handshake among themselves: to convey trust, competence, and the openness to interact. We need to develop guidelines that captures how we can mimic human handshakes for humanoid robots. Trust needs touch.

There is an infinite set of paths connecting two points in space in a handshake. Nelson (1983) observed that when a person moved his or her hand between two points, the person’s central nervous system selects only one path out of the infinite possible paths. He suggested that dynamic models alone cannot describe the performed movement. Dynamic models relate the movement to the applied muscle forces but do not account for the signals from the central nervous system to the muscles. In other words, the central nervous system makes a choice that ultimately affects the path selected. This led to a number of optimization principles that were intended to aid the dynamic models. These principles add an extra constraint on the path to be selected to account for or to model the choice that the central nervous system executes. Nelson (1983) further described the optimization principles that minimized the movement time, exerted energy, and jerk (i.e., the first derivative of acceleration). Moreover, he examined the possibility of a compromise between the models. The jerk minimization model by Hogan (1984) was based on single-joint movements with large amplitudes and moderate speeds. Flash and Hogan (1985) further expanded the model to predict two joints and *via*-point movements. Edelman and Flash (1987) implemented this model for two-dimensional handwriting movements.

This paper aims to extract the nuances of human-to-human handshake movement for a humanoid robot to replicate. With that aim having been achieved, this work contributed the following:

1) The phases of human-human handshake movements were determined. The full handshake movement consists of three main phases: reaching, contact, and return. Contrary to the common notion that the contact phase consists only of the up and down handshake, we showed the following subphases of the contact phase: preshake, handshake, postshake, and no motion.
2) The proposed multiphase jerk minimization model was able to replicate the smooth motion of the arms when two humans shake their hands. The model allowed us to calculate displacements, velocities, and accelerations that enabled us to better understand the multiphase nature of the handshake.
3) Our work demonstrated a complete motion of a handshake when compared with the other prevalent handshake models. An initial step in achieving a believable and humanlike handshake behavior by a humanoid is achieving a realistic handshake that has similarities to the basic motion patterns.
4) The guidelines for achieving a more natural human-robot handshake were proposed. There are many nuances in human-to-human handshake and the guidelines provide insights on how lifelike human-robot handshakes can be achieved.

This paper is organized as follows. The next section presents some of the earlier works in modeling handshakes. Section 3 describes the design and procedures for the human-to-human handshake experiments. Section 4 describes the division of the handshake process into three main phases. Section 5 shows the mathematical modeling for the handshake movement and the results are presented in Section 6. Section 7 provided the analysis and discusses the guidelines for human-robot handshake. Finally, Section 8 concludes the study and gives the recommendations for future work.

## 2 Related Works

### 2.1 Modelling the Handshake Phases

Kasuga and Hashimoto (2005) implemented neural oscillators to simulate the movement of the robot in physical human-robot interaction. The neural oscillator made use of the synchronization of movements during the interactions. In this approach, the applied forces and torques were used as inputs to determine the trajectory of the shoulder and elbow joints of a robot arm. By adjusting the parameters of this system, the passive behavior of the robot’s handshake was adjusted. From computer simulations and experimental validation, results showed that the proposed neural oscillator control method was better than the conventional impedance control method.

Extending the work of Kasuga and Hashimoto (2005), Jindai et al. (2006) proposed the use of a second-order lag element to simulate the reaching movement of the handshake receiver. The proposed reaching movement was a weighted combination of the reaching movement of the initiator and a following movement of the initiator’s hand. The value of the weighting coefficient changed as the movement progressed such that the movement switched from the former to the latter. A handshake experiment was performed to evaluate the proposed method. The velocity plots of the reaching movement of the receiver and the one obtained by applying this method were similar, which showed the acceptability of the procedure.

Yamato et al. (2008) proposed a preshaking movement to add to the reaching movement proposed by Jindai et al. (2006) and to the handshaking movement proposed by Kasuga and Hashimoto (2005). However, this led to modifying the model of the reaching movement by adding a dead-time element to the transfer function. The proposed preshake movement was hypothesized to exist as a leading initiative by the receiver to start the handshake. This movement was simply a quarter of a handshake cycle that was directed upward or downward, or was absent (i.e., the initiator led the shaking movement). These three conditions were tested in a second handshake experiment between the participants and a robot system. The authors reported that the upward preshake movement was the most acceptable of the three. A third experiment was conducted to determine the best out of the three models: the selected upward model, a height-based movement direction model, and a force direction model. The height-based model selected the direction of the movement based on the height at which contact was made. The force direction model moved in the direction that matched the direction of the force applied by the handshake initiator. The height-based model was reported to be the best out of the three models.

Jindai et al. (2012) proposed a model for the reaching movement of the handshake initiator. The proposed model was based on a combination of fast and slow movement instances of Hogan (1984)’s model. This approach was utilized to obtain a skewed bell-shaped velocity profile, which was the reaching movement profile. A handshake experiment between the participants and a small robot system was conducted to determine the best value of the parameters of the lag and dead-time elements of the control system. It was determined that the receiver's movement that lagged behind the initiator’s movement and incorporated a dead-time period was preferred.

In Wang et al. (2008), they proposed a model for the handshaking contact wherein the modelling procedure started from acquiring numerous handshake data. Then, the desired trajectories were generated by a planner and were synthesized by a motion synthesis module. Finally, new control methods were employed to achieve a satisfactory robot handshake. The controller design made use of force-based impedance control.

In Tagne et al. (2016), they studied handshake in the context of various greetings (i.e., hello, congratulations, sympathies). They found that the context has an effect on the strength and duration of the handshake. Similarly in Melnyk and Henaff (2019), they found that there is a difference in the duration of handshake in the context of handshake for greetings and that for consolations.

### 2.2 Leader and Follower Synchronization Methods

Avraham et al. (2012) proposed three handshake leader and follower methods: the Tit-for-Tat model, *λ* model, and iML-Shake model. The Tit-for-Tat model was based on the assumption that the shaking movement involved a leader and a follower. The leader imposed his/her handshake style while the follower conformed to and imitated it. Thus, the Tit-for-Tat model involved recording and repeating the shaking movement of the leader. This required either prior knowledge of the shaking movement (when taking the leader’s role) or recording a portion of the movement and repeating or following it (when taking the follower’s role).

The *λ* model was based on the assumption that the hand alternates between two threshold positions during the handshaking movement and the smoothness of the resulting path is a result of the physiology and biomechanics of the muscles. This model required instantaneous data of the participant’s hand position, an understanding of the biomechanics of the arm movement, and an estimation of movement parameters.

The iML-Shake model is a machine-learning model that was based on the concept that human movements can be viewed as a function that takes positions as inputs and produces forces as outputs. The model required the collection of movement data and performing linear regression to estimate the parameters of the model. The position of the interrogator’s hand was also required when performing the handshake test. The Tit-for-Tat and the iML-Shake models were considered to be more humanlike than the *λ* model. The authors suggested developing a model that captured the advantages of all of the proposed models.

Jouaiti et al. (2018) introduced the Hebbian plasticity in central pattern generator controllers to facilitate self-synchronization for human-robot handshaking. With this mechanism, they showed that the synchronization had a transitory phase and the permanent phase. In the transitory phase, the system adapts and learns the handshake conditions while in the permanent phase, the system retained the learning.

Mura et al. (2020) proposed a handshake robot system that implemented an extended Kalman filter (EKF) to learn from a human handshaking partner and to mimic the handshake. The EKF was designed to observe the intention of the human and turn this into an appropriate control reference for the robot arm. For the handshake movement, a sinusoidal function of unknown time-varying amplitude and frequency were implemented. The robot arm and hand system was able to synchronize with the human motion and to anticipate an active, leading behavior.

### 2.3 Summary

The models proposed in Kasuga and Hashimoto (2005); Jindai et al. (2006); Yamato et al. (2008); Wang et al. (2008) and the Tit-for-Tat model (Avraham et al., 2012; Mura et al., 2020) were based on collecting movement data from the handshake partner. This approach leads to replicating or imitating the performed movement and removes any differences in handshake characteristics that normally exist between two individuals who are performing a handshake. Moreover, in certain situations, a warm greeting (e.g., when welcoming a guest) or a firm one (e.g., in an interview) is expected from one side.

Without the ability to collect the movements from the handshake partner, these types of handshakes will be impossible to perform. The iML-shake model proposed in Avraham et al. (2012) introduced a machine learning strategy whereby the participants’ behavior data related to their positions and grasping forces were used as an input-output function of their algorithm. According to the authors, it assumed little about the handshake movement, which made it more useful, because any unknown biomechanical features will be included. However, this feature is its main weakness. Ideally, a model like the *λ* model (Avraham et al., 2012) should be sought such that the important features of a movement are included and the weak or random influences are removed.

The reason this model did not perform that well could be due to the observation of Nelson (1983) wherein he stated that dynamic models are not enough to describe human movement alone and a form of optimization should be sought to compliment them. This approach was utilized in developing the model proposed in Jindai et al. (2012) by building on Hogan’s jerk minimization model (Hogan, 1984). The present paper builds on these results to develop an improved model for a smooth human-to-human handshake, which can then be replicated in human-robot handshakes.

## 3 Handshake Experiments

### 3.1 Participants

An experimenter was selected to wear a set of motion and force sensors and was trained to perform the handshakes. Due to the long duration required for wearing and calibrating the sensors and the training time for the experimenter, only one participant played the role of the experimenter. Another five participants were selected to shake hands with the experimenter, who were naive about the hypothesis of the research study. All the participants selected were right-handed males and their hand dimensions were close to the 50th percentile as compared to the anthropometrical dimensional data [i.e., 19.3 cm hand length, 8.9 cm width; (Man-Systems Integration Standards, 1995)].

The data of the experimenter and participants are summarized in Table 1. They did not report any neuromuscular or neurological disease that might interfere with motor function. Approval for the handshake experimental protocol was granted by the Institutional Review Board of the National University of Singapore and the procedures involved in this study were in accordance with the World Medical Association’s Code of Ethics (Declaration of Helsinki). Data were stored and analyzed anonymously. All participants gave their written informed consent.

**TABLE 1:** Experimenter and Participants Data.

	Experimenter	Participant Mean (SD)
Age (years)	22	25 (0.75)
Height (cm)	175	174 (2.51)
Weight (kg)	80	77 (5.73)
Hand length (cm)	19.3	19.3 (0.19)
Hand width (cm)	8.9	8.8 (0.18)

### 3.2 Experimental Design

**FIGURE 1:**
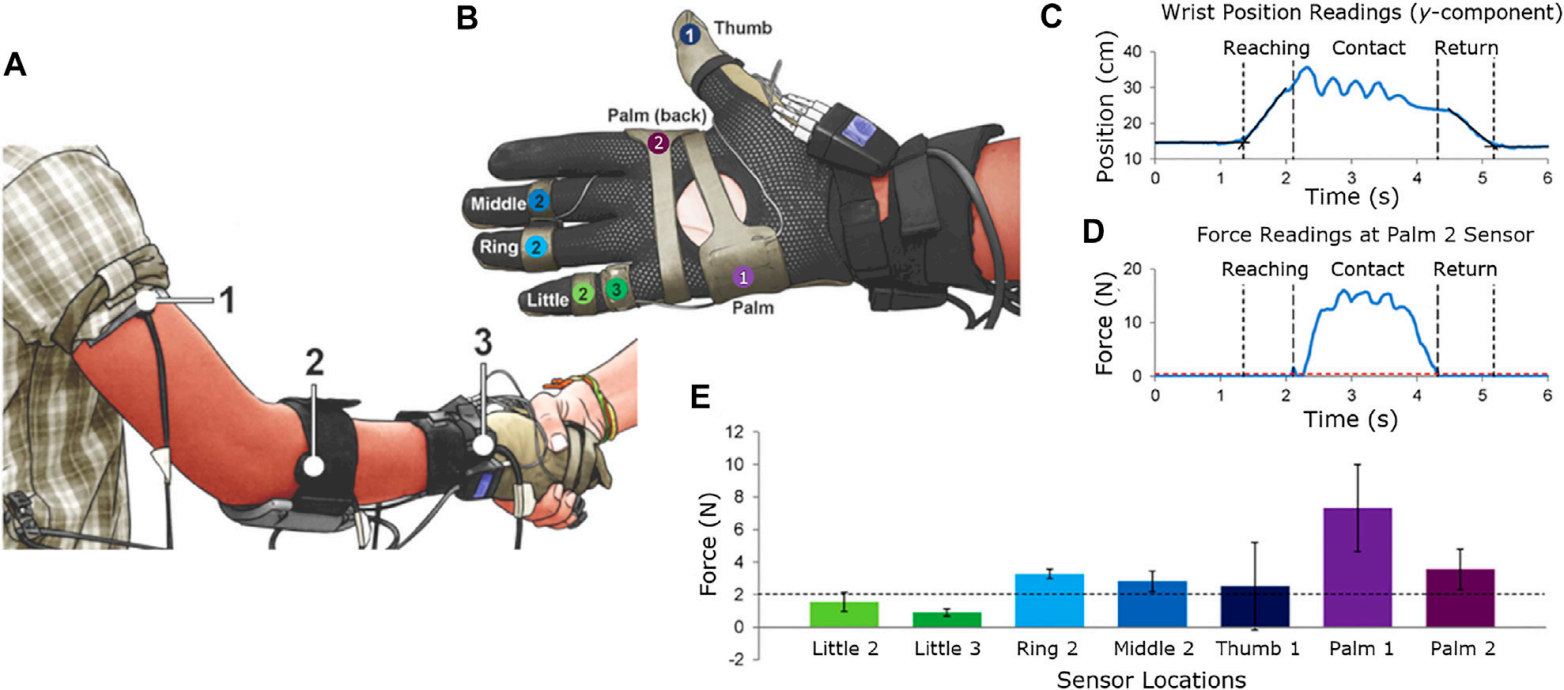
Locations and numbering of the motion tracking sensors. **(A)** Sensors 1, 2, and 3 are located on the upper-arm below the shoulder joint, on the forearm below the elbow joint, and on the wrist joint, respectively. **(B)** Locations and average readings of the force sensors and determination of movement and contact durations. The locations of the force sensors are shown. **(C,D)** The procedures for determining the start and end of movement and contact for the first dataset are shown. **(E)** The mean contact forces of each force sensor.

**FIGURE 2:**
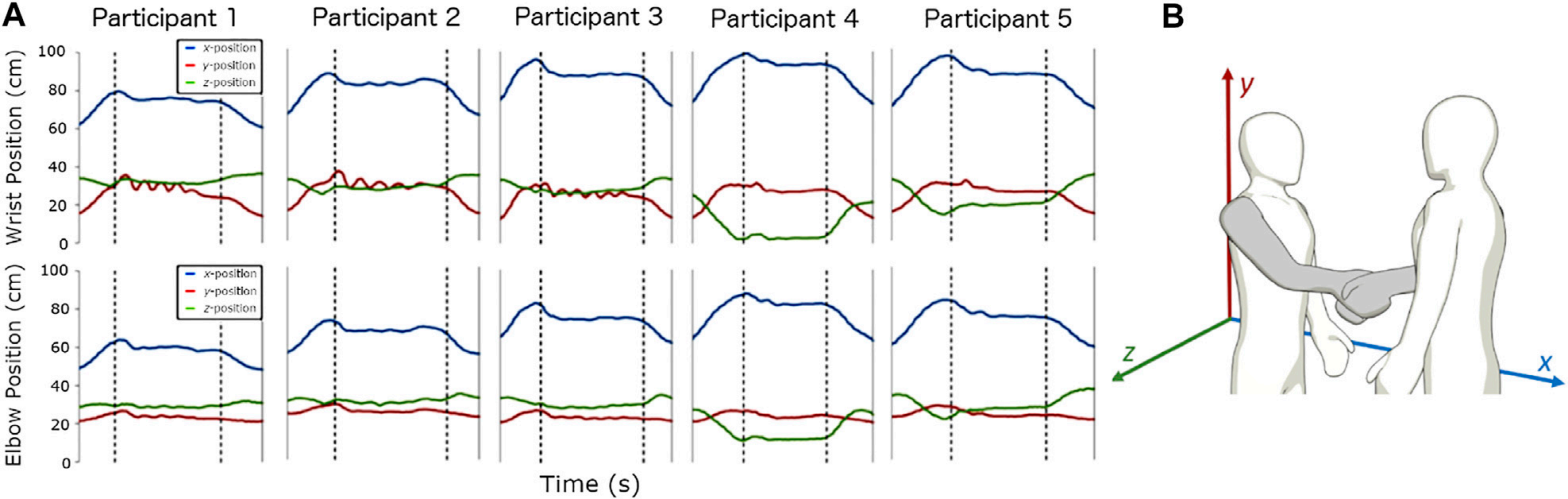
Position plots and description of the position of the hand. **(A)** Shown are the plots from the sensors at the wrist and at the elbow of the participants. **(B)** The location of the transmitter of the tracker, which serves as the reference frame for the tracker data. The *x*-position (blue) represent the reaching, *y*-position represent the up and down arm movements, and the *z*-position represent the panning adjustments to the handshake partner.

There is substantial benefit in replicating human motion to inspire behaviors in robots (Kulic et al., 2012; Maeda et al., 2017; Cini et al., 2019). In such approaches, visual trackers and sensors are often employed to capture and learn from human movements. In the present work, the experimenter wore two sets of sensors. Motion trackers (Fastrak, Polhemus Inc, VT, United States) were mounted proximally to the shoulder, elbow, and wrist joints (Figure 1A). The trackers recorded the 3D position in the *xyz* axes of each sensor relative to transmitter of the trackers (Figure 2A). The transmitter was positioned on top of a desk behind the experimenter and serves as the reference frame for the motion trackers. Figure 2B shows the experimenter and participant in the positive *xyz* plane.

Wearable force sensors (Finger TPS, Pressure Profile Systems, CA, United States) were also worn to measure the normal contact forces exerted during the handshake. The force sensors were positioned on seven locations: the middle phalanges of the middle, ring, and little fingers, the proximal phalange of the little finger, the distal phalange of the thumb, and the volar and dorsal sides of the hand, as shown in Figure 1B. These locations were selected because these are the locations with high skin compliance and where the maximum handshake contact forces occur (Cabibihan et al., 2006a, 2011). The motion trackers and force sensors have a sampling rate of 30 Hz.

With the experimenter trained to give a neutral handshake, he was further instructed to keep his grasping force to a minimum while allowing the receiver to apply his natural handshake grasp and motion. This was similar to the protocol in Chaplin et al. (2000) where they determined the relationship between handshakes and personality. In a neutral handshake, the experimenter extended his right hand to initiate a handshake. Upon contact with the experiment participant, the experimenter grasped the other person’s hand, but waited for the other to initiate the grip and the upward and downward shaking. Moreover, the experimenter was instructed to relax the grasp once the partner relaxed his grasp.

The participants were introduced to the experimenter and were told to respond naturally to the experimenter’s handshake. The experimenter and each participant were instructed to stand face-to-face within 1 m from each other. After starting the data acquisition, the experimenter was instructed to initiate the handshake. Data acquisition continued for at least 1 min after finishing the handshake. Each participant performed only one handshake with the experimenter to avoid any learning effects that could influence the data, such as those reported in Jindai et al. (2006).

### 3.3 Data Analysis

The human upper limb consists of the hand, forearm, and upper arm with 7 DOFs that are made possible by the wrist, elbow, and shoulder joints (Kim et al., 2011). The pronation/supination of the hand, which originates from the forearm (Taylor and Schwarz, 1955), and the fine finger movements were not investigated in this experiment since those require separate analysis. Analyses were done on the contact forces recorded for each sensor location, differences in the handshake phases in human-human handshakes, and differences among the various handshake models.

When the hand is grasped during handshakes, the forces on the fingers oppose the forces on the palm. We expect that the sum of the normal forces exerted by the fingers is equivalent to the sum of the normal forces exerted by the palm to counterbalance the forces. We want to determine whether the selected locations of the sensors were appropriate. If our sensor placements are correct, the sum of the normal forces must be equal to zero where the normal forces from the fingers are equal and opposite to ones on the palm (Eq. 1):

$$\overset{5}{\underset{f=1}{\sum }}F_{f}=\overset{2}{\underset{p=1}{\sum }}F_{p}$$ (1)

where the subscript *f* denotes the sensors positioned on the fingers and *p* denotes the sensors on the palm (Figures 1B,E).

To determine whether there was a difference among the handshake phases, the two-sample t-test has been conducted between all possible combinations of the phases. However, this only has been used for the ones that could be assumed to have equal variances. Moreover, Welch’s t-test has been used for the non-equal variance. Furthermore, the *F*-test has been first used to test if the assumption of the equal variance could be applied or not. We found that the assumption of equal variances could be applied only between the reaching and return phases based on the conducted *F*-test. That indicates that the two-sample *t*-test (equal variance t-test) would be used to investigate the mean difference between reaching and return phases, while the others would use Welch’s *t*-test (non-equal variance t-test). All statistical analyses were performed using a statistical package (Minitab v17, Minitab, LLC, PA, United States). A *p*-value of ≤0.05 was considered statistically significant. Furthermore, our model was compared with other handshake models in earlier physical human-robot interaction works. Only the contact phase was similar among them. Thus, the contact phase was compared using the percentage difference equation.

## 4 Handshake Phases

Two procedures were developed to divide the handshake into phases. The first distinguished *movement* from *no movement*. The second distinguished *contact* from *no contact*. The vertical component (*y*-position) of the sensor at the wrist (Figure 1C) was used to identify the start and end of movement. Next, lines were fitted to the data before and after the start and end of movement. At that point, four lines were obtained: two lines for the increasing and decreasing slopes and two for the zero movement (Figures 1C,D). Finally, the points of intersection of the lines were determined. The time components of these points indicated the start and end of the handshake movement.

Figure 1E shows the grasping normal forces during handshake. Table 2 shows the results of the statistical differences between each pair of locations where the force sensors were mounted. These tests were necessary to test the validity of the handshake grasp. The values shown are the *p*-values that resulted from the two-sample t-test (equal variance t-test) and the Welch’s t-test (non-equal variance t-test). The sensors with *p*-values less than 0.05 indicate that there is a significant difference between the two sensors that were compared. Moreover, the *F*-test was conducted before the t-test analyses in order to investigate the variance equality and determine which t-test would be used. For example, Little2 and Little3 have contact normal forces that are not significantly different from one another. However, Little2 and Ring2 are significantly different (Figure 1E). It can also be observed that the sensor at the Palm1 location was significantly different from all the sensors at the 6 other locations.

**TABLE 2:** Statistical differences between the normal contact force sensors in various locations of the hand (*p*-values).

	Little2	Little3	Ring2	Middle2	Thumb1	Palm1
Little3	0.052	—	—	—	—	—
Ring2	<0.001*	<0.001*	—	—	—	—
Middle2	0.011*	<0.001*	0.185	—	—	—
Thumb1	0.603	0.411	0.673	0.861	—	—
Palm1	0.009*	0.006*	0.028*	0.021*	0.049*	—
Palm2	0.012*	0.009*	0.649	0.275	0.470	0.021*

* Significant difference at *p* = 0.05.

To further verify whether the locations of the sensors were correct in Figure 1E, we performed a force balance calculation in Eq. 1. The sum of the mean forces from the fingers was 11.03 N while the mean forces from the palm was 10.88 N. The percentage difference was 1.39%. This implies that handshake grasp was stable. The forces that were unaccounted for may be due to locations where additional sensors are needed.

The start time of contact is the time when at least one sensor started reading non-zero contact forces. The end time of contact is the time when all the sensors stopped reading contact forces. The determination of the start and end time of movement and contact was done for all the participants’ data. Using the start and end time of movement and contact, the durations of the handshake phases were determined (Table 3).

**TABLE 3:** Duration of the phases of the handshake (in sec).

Phase	Participant 1	Participant 2	Participant 3	Participant 4	Participant 5	Mean (SD)
Reaching	0.76	0.94	0.99	1.01	0.89	0.92 (0.10)
Contact	2.20	2.16	2.47	1.58	1.37	1.96 (0.46)
Return	0.86	0.61	0.70	0.89	0.68	0.75 (0.12)
Total	3.82	3.71	4.16	3.48	2.94	3.63 (0.45)

The *xyz*-position readings of the sensors at the wrist and at the elbow were relevant to the description of the hand movement (Figure 2). There are three main phases during a handshake movement: arm reaching, hand contact, and arm return. The reaching phase is the first phase of the handshake where the handshake initiator extends his/her hand. The contact phase is the phase where contact occurs between the two hands and was distinguished in this experiment with the force sensors. The return phase is the last phase of the handshake where the handshake initiator retracts the hand to its original position. Noticeable in the wrist and elbow movements were the non-uniform contact phase among the participants.

Once contact was detected and a stable grasp was made, the oscillating handshake movements began. It appears that the features of the handshake movement were a result of the characteristics of the initiator’s handshake as well as the receiver’s. Different handshake movements were observed, even though only one side of the handshake (i.e., the experimenter’s hand) was collected. After the handshake movement was completed, there was a short duration where contact still existed, but no motion was detected. During the return phase, the hand moved backward and downward towards its neutral initial position. The duration of this phase was shorter than the duration of the reaching phase.

On average, the handshakes lasted for 3.63 s (SD = 0.45 s) and can be divided into: 0.92 s (SD = 0.10 s) for the reaching phase, 1.96 s (SD = 0.46 s) for the contact phase, and 0.75 s (SD = 0.12 s) for the return phase (Table 3). The reaching, contact, and return phases corresponded to 25, 54, and 21%, respectively, of the whole handshake movement. Significant difference was found between the contact and reaching phases; *t*(4) = 4.92, *p* = 0.008. There was also a significant difference between the reaching and return; *t*(8) = 2.42, *p* = 0.042. Furthermore, there was a significant difference between the contact and return phases, *t*(4) = 5.67, *p* = 0.005. The pairwise t-test comparisons confirm that each phase is statistically distinct from one another in terms of the phase duration. In other words, this test confirmed that the reach and return phases are not simple inversions of one another in timing. This insight provided additional justification on why each phase needed to be investigated.

## 5 Modelling

The handshake movement can be fully described when its position is provided for the duration of the movement. We assumed that the wrist moved in a single plane (i.e., *xy*-plane; Figures 1A, 2B). According to Flash and Hogan (1985), “unconstrained point-to-point motions” involve almost straight paths. Since the reaching and return motions are of that type, and that the motion of the contact phase was mostly along the vertical direction, the assumption was justified. A similar assumption was done in Kasuga and Hashimoto (2005). Likewise, Mura et al. (2020) restricted their dynamic system to a 1 DOF case, where the handshake motion occurs only in the vertical plane.

There are three directions of the hand position according to the *xyz*-directions (Figures 1A, 2A). Henceforth, the direction along the reaching motion of the hand is referred to as the horizontal *x*-position. The plane perpendicular to the ground is referred as the vertical *y*-position, which also represents the up and down movement of the handshake. The panning movement (i.e., *z*-direction) was neglected due to the relatively low changes in its values.

All the models of the phases and subphases, with the exception of the handshaking motion model, were based on the jerk-minimizing fifth degree polynomial proposed by Hogan (1984). This approach was recommended as it is appropriate for point-to-point natural human motions (Flash and Hogan, 1985). Hogan’s method was similar to the data fitting spline interpolation technique, where a spline is one or more polynomial functions, which is set up to provide a smooth transition between two or more points under some conditions (Chapra, 2012). The spline interpolation technique provided identical results to Hogan’s method when two data points were fitted to a quintic spline under the conditions that the first and second derivatives of the resulting polynomial are zero at these points. The total number of conditions is six where passing the points amounted to two while at the end points, the zero first and second derivatives amounted to four. This allowed us to use Hogan’s method under different conditions (Supplementary Appendix).

### 5.1 Vertical Position Model

The motion was divided into three main phases. We first developed the model for the contact phase because this was the commonly modeled phase for handshakes. After that, a different model was used for both the reaching and return phases.

#### 5.1.1 Contact Phase

Figures 1C,D and 2A showed the contact phase involving shaking, an oscillatory motion, as well as a region with little or no motion at the end. The oscillatory motion was observed to have an exponential decay. These observations led to a model in the form of (Eq. 2):

$$y_{\text{handshake}}=A_{V1}e^{-\gamma t}\cos\left(\omega t+\phi \right)-A_{V2}t+A_{V3}$$ (2)

where $\cos\left(\omega t+\phi \right)$ describes the oscillatory nature of the response, *e* ^−*γt*^ represents the exponential decay, and −*A*_*V*2_ *t* represents the downward trend. The variables *γ*, *ω*, *ϕ*, *A*_*V*1_, *A*_*V*2_, and *A*_*V*3_ are the decay constant, the angular frequency, the phase shift, the amplitude at time zero, the downward trend slope, and the intercept, respectively.

The contact phase was further divided into three subphases: preshake, main shake, and postshake. The pre- and postshake subphases were hypothesized to exist as smooth transitions from and to the static equilibrium states at the onset and at the termination of the shaking subphase. Thus, only the middle portions (main shake subphase) of the datasets were fitted to Eq. 2. The pre- and postshake subphases can be described using fourth degree polynomials (Eqs 3,4):

$$y_{\text{preshake}}=B_{V4}t^{4}+B_{V3}t^{3}+B_{V2}t^{2}+B_{V1}t+B_{V0}$$ (3)

$$y_{\text{postshake}}=C_{V4}t^{4}+C_{V3}t^{3}+C_{V2}t^{2}+C_{V1}t+C_{V0}$$ (4)

**FIGURE 3:**
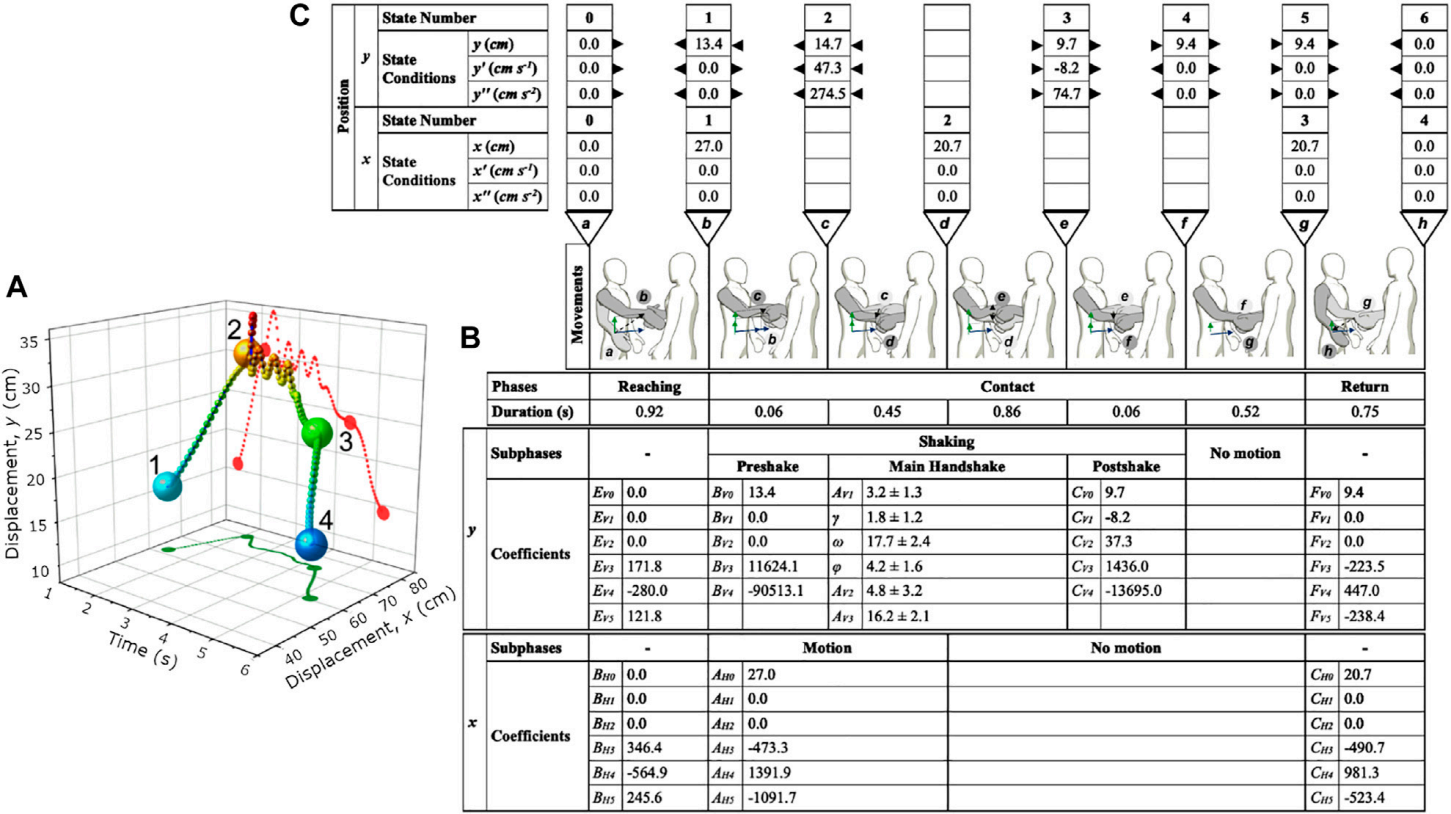
Visualization of the position sensor at the wrist during a handshake and the modeling parameters. **(A)** Representative result from a single handshake trial. The big dots 1, 2, 3, and 4 represent the start and end points of the three phases: reaching, contact, and return phase. **(B)** Illustrations of the handshake movements, phases, durations, and subphases. The phases and subphases of the handshake are listed together with the calculated model coefficients for *y* and *x* positions. The mean and standard deviation are shown only for the relevant *y* wrist position. **(C)** The states and state conditions of *y* and *x* positions, respectively, are listed and numbered. The arrows next to the *y*-position conditions indicate whether the value was determined from the phase in-between the states as an output or was used as a condition for the determination of the phase model coefficients. For the *y*-coordinate position, all the state conditions originated from the main shake subphase since it was the first vertical motion modeled. For *x*-coordinate position, all the state conditions were established beforehand, so the models of the phases and subphases can be determined in any order.

The conditions for Eq. 3 were tabulated in Table 4. Moreover, the coefficients and *y*_1_ have been calculated using Supplementary Appendix Equations SA4, SA11 in the Supplementary Appendix SA. The conditions for Eq. 4 were tabulated in Table 5. Subsequently, the variables *y*_0_ to *y*_6_ are the State Numbers found in the top row of Figure 3C.

**TABLE 4:** Preshake subphase conditions (vertical movement).

Static equilibrium state (state 1)	Static equilibrium state (state 2)
*y* (0) = *y*_1_ → unknown	*y* (*D*_*V*2_) = *y*_2_
*y*′(0) = 0	$y^{\prime }\left(D_{V2}\right)=y_{2}^{\prime }$
*y*′′(0) = 0	$y^{\prime \prime }\left(D_{V2}\right)=y_{2}^{\prime \prime }$

**TABLE 5:** Postshake subphase conditions (vertical movement).

Static equilibrium state (state 3)	Static equilibrium State (state 4)
*y* (0) = *y*_3_	*y* (*D*_*V*4_) = *y*_4_ → unknown
$y^{\prime }\left(0\right)=y_{3}^{\prime }$	*y*′(*D*_*V*4_) = 0
$y^{\prime \prime }\left(0\right)=y_{3}^{\prime \prime }$	*y*′′(*D*_*V*4_) = 0

The final handshake state was obtained by substituting time, *t*, which was the average main shake duration in the handshake model. The coefficients and *y*_4_, which was initially unknown, were calculated using Supplementary Appendix Equations SA4–SA9, and Supplementary Appendix Equations SB1, SB2. These were derived in the Supplementary Appendix SA, SB.

#### 5.1.2 Reaching and Return Phases

Fifth degree polynomials (Eqs 5, 6) were used to model the reaching and return phases because the number of conditions for each was six:

$$\begin{array}{c} y_{\text{reaching}}=E_{V5}t^{5}+E_{V4}t^{4}+E_{V3}t^{3}+\\ E_{V2}t^{2}+E_{V1}t+E_{V0} \end{array}$$ (5)

$$\begin{array}{c} y_{\text{return}}=F_{V5}t^{5}+F_{V4}t^{4}+F_{V3}t^{3}+\\ F_{V2}t^{2}+F_{V1}t+F_{V0} \end{array}$$ (6)

The conditions of the reaching phase are shown in Table 6. *D*_*V*1_ is the mean duration of reaching phase of the vertical movement. For the return phase, the conditions are shown in Table 7. *D*_*V*6_ is the mean duration of return phase of the vertical movement. The coefficients in Eqs 5, 6 were calculated using Eqns. C4 to C12 (Supplementary Appendix SC).

**TABLE 6:** Reaching phase conditions (vertical movement).

Static equilibrium state (state 0)	Static equilibrium state (state 1)
*y* (0) = *y*_0_	*y* (*D*_*V*1_) = *y*_1_
*y*′(0) = 0	*y*′(*D*_*V*1_) = 0
*y*′′(0) = 0	*y*′′(*D*_*V*1_) = 0

**TABLE 7:** Return phase conditions (vertical movement).

Static equilibrium state (state 5)	Static equilibrium state (state 6)
*y* (0) = *y*_5_	*y* (*D*_*V*6_) = *y*_6_
*y*′(0) = 0	*y*′(*D*_*V*6_) = 0
*y*′′(0) = 0	*y*′′(*D*_*V*6_) = 0

### 5.2 Horizontal Position Model

Unlike the vertical motion, the horizontal motion was consistent across the datasets. Thus, the mean values for the key states and durations of the phases were obtained and were used in the modelling process.

#### 5.2.1 Contact Phase

The contact phase involved movements only during its initial portion in the *x*-direction (horizontal position model), as most of the shaking movements in this phase were in the *y*-direction (vertical position model), as shown in Figure 2A. The model of the contact phase is in the form of Eq. 7:

$$\begin{array}{c} x_{\text{contact}}=A_{H5}t^{5}+A_{H4}t^{4}+A_{H3}t^{3}+\\ A_{H2}t^{2}+A_{H1}t+A_{H0} \end{array}$$ (7)

The conditions of this motion are shown in Table 8. *D*_*H*2_ is the mean duration of the motion subphase of the contact phase of the horizontal movement. The coefficients were calculated using Supplementary Appendix Equations SC4–SC9 (Supplementary Appendix SC). The variables *x*_0_ to *x*_4_ are the State Numbers found in the fifth row from the top in Figure 3C.

**TABLE 8:** Motion subphase conditions (horizontal movement).

Static equilibrium state (state 1)	Static equilibrium state (state 2)
*x* (0) = *x*_1_	*x* (*D*_*H*2_) = *x*_2_
*x*′(0) = 0	*x*′(*D*_*H*2_) = 0
*x*′′(0) = 0	*x*′′(*D*_*H*2_) = 0

#### 5.2.2 Reaching and Return Phases

The reaching and return phases of the horizontal motion were treated in the same manner as the reaching and return phases of the vertical motion. Their models were in the form of Eqs 8, 9, respectively:

$$\begin{array}{c} x_{\text{reaching}}=B_{H5}t^{5}+B_{H4}t^{4}+B_{H3}t^{3}+\\ B_{H2}t^{2}+B_{H1}t+B_{H0} \end{array}$$ (8)

$$\begin{array}{c} x_{\text{return}}=C_{H5}t^{5}+C_{H4}t^{4}+C_{H3}t^{3}+\\ C_{H2}t^{2}+C_{H1}t+C_{H0} \end{array}$$ (9)

The conditions of the reaching phase are shown in Table 9. *D*_*H*1_ is the mean duration of the reaching phase of the horizontal movement. Similarly, the conditions of the return phase are shown in Table 10. *D*_*H*4_ is the mean duration of the return phase of the horizontal movement. The coefficients of Eqs 7, 8 were calculated using Supplementary Appendix Equations SC4–SC12 in Supplementary Appendix SC.

**TABLE 9:** Reaching phase conditions (horizontal movement).

Static equilibrium state (state 0)	Static equilibrium state (state 1)
*x* (0) = *x*_0_	*x* (*D*_*H*1_) = *x*_1_
*x*′(0) = 0	*x*′(*D*_*H*1_) = 0
*x*′′(0) = 0	*x*′′(*D*_*H*1_) = 0

**TABLE 10:** Return phase conditions (horizontal movement).

Static equilibrium state (state 5)	Static equilibrium state (state 4)
*x* (0) = *x*_3_	*x* (*D*_*H*4_) = *x*_4_
*x*′(0) = 0	*x*′(*D*_*H*4_) = 0
*x*′′(0) = 0	*x*′′(*D*_*H*4_) = 0

## 6 Results

We will first provide a summary of the modelling results and then describe the handshake kinematics, comparisons between the model and experiments, and the comparisons between the model and other handshake models.

### 6.1 Handshake States, Phases, and Coefficients

The displacement data from the sensor at the wrist was plotted in a three-dimensional graph (Figure 3A). Only the displacements for the *x*-coordinate position (i.e., horizontal movement) and the *y*-coordinate position (i.e., vertical movement) were considered. The panning movement (i.e., *z*-coordinate position) had a small change with respect to time and was thus neglected in our analysis. Moreover, our own experiences tell us that excessive movements along the *z*-plane during the handshake oscillation is not usual.

From Figure 3A, it can be observed that there is a rapid rate of increase in the vertical movement during the reaching phase (14.16 cm s^−1^). During the contact phase, the vertical displacement changed to a damped oscillation. There can be around 2-4 oscillations during handshake. Lastly, there was a slower rate of decrease in the vertical displacement during the return phase to complete the handshake (−9.78 cm s^−1^).

The states, phases, durations of phases, subphases, calculated coefficients of the models, and illustrations of the states and phases were summarized. For ease of understanding, the reader is referred to the handshake phases in Figure 3B, which consists of the reaching, contact, and return. The *y*-coordinate position and *x*-coordinate position models were further presented with the summary of the coefficients. Both models have a reaching phase at the start and return phase at the end. The contact phase can be further divided into subphases. For the *y*-coordinate position, the contact phase has preshake, main shake, postshake, and no motion subphases. For the *x*-coordinate position, the contact phase has motion and no motion subphases.

Figure 3C shows the state conditions for the displacement, velocities and accelerations for the *y* and *x*-coordinate positions. An arrowhead pointing outward indicates that the data were obtained from the phase (i.e., these served as outputs to other phases as conditions). An arrowhead pointing inward to the column of numbers indicates that the data were used to determine the phase (i.e., as state conditions).

### 6.2 Handshake Kinematics

The hand movement models provided a description of the handshake kinematics. During the reaching phase (State 1), the wrist moved from its default position at the hips to a position of about 13.4 cm upward and 27 cm forward (Figure 3C). Once contact with the other hand was detected, there was an increase of around 1.3 cm [State 2 (14.7 cm)–State 1 (13.4 cm)] to reach the peak of the *y*-coordinate displacement of the handshake. In other words, before the main handshake occurs, the hands are first raised to the peak vertical values before the handshake oscillations commence. During the contact phase, the hand accelerated and started the handshaking movement while the hand relaxed slightly backward to −6.3 cm [subphase d (20.7 cm)–subphase b (27.0 cm)]. This meant that a position correction was done from the initial overshoot of the reaching movement.

**FIGURE 4:**
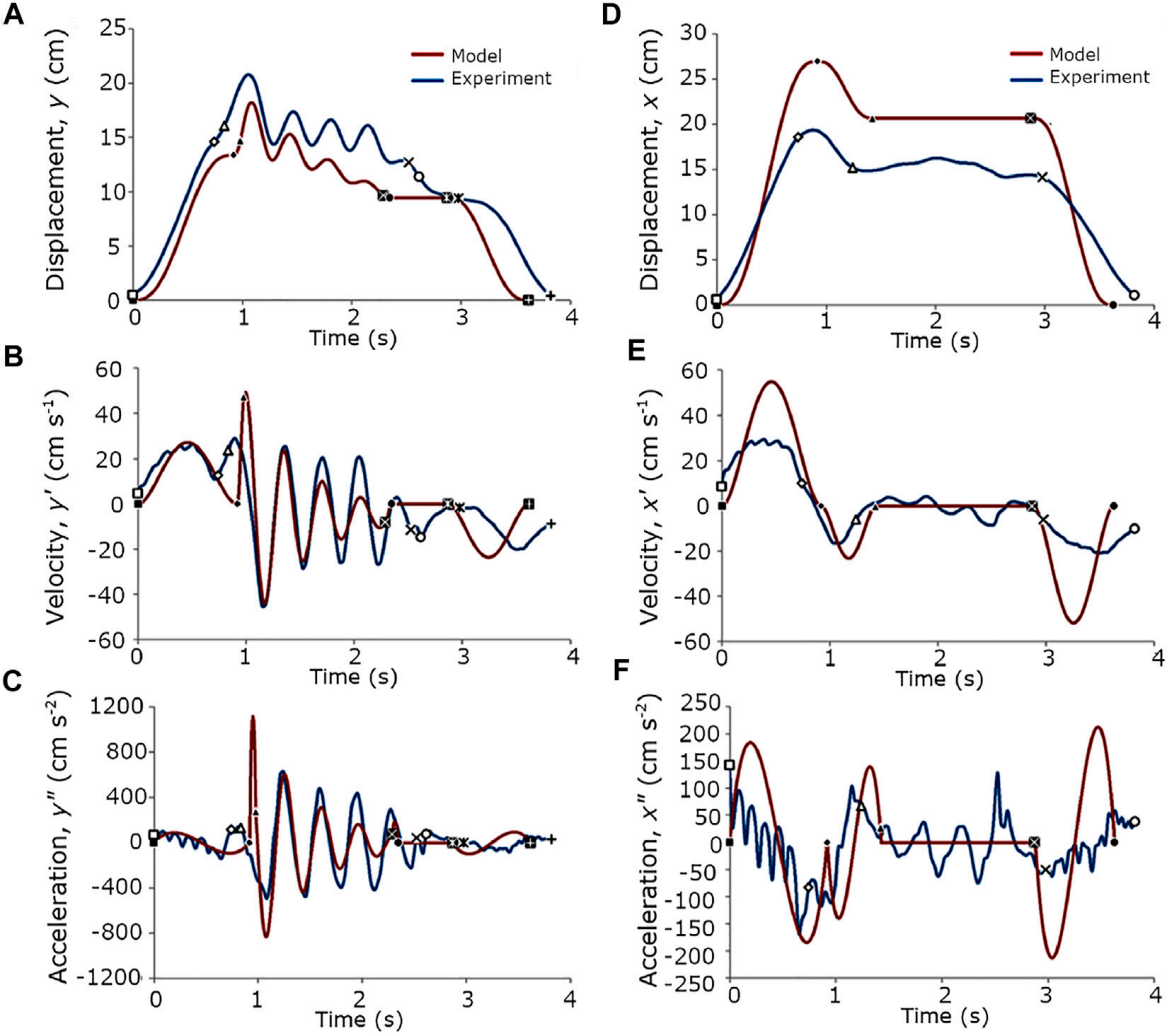
Comparisons between the model and experiments. **(A–C)** *y*-axis position, velocity, and acceleration, respectively. **(D–F)** *x*-axis position, velocity, and acceleration, respectively. The experiment curves came from the mean of the collected data.

The handshaking motion decreased in amplitude as it progressed with about three to four oscillations (Figures 3A, 4A). The hand decelerated to a stop to about 5 cm [State 2 (14.7 cm)–State 3 (9.7 cm)] lower than its position at the start of the handshake. Moreover, the handshaking motion was mainly a feature of the vertical hand movement. This was shown on the *y* and *x* axis displacement results. During the return phase, the hand moved downward (Figure 4A) and backward (Figure 4D) towards its initial neutral position, which cleared all the grasping forces and movements that remained after contact (also refer to Figures 1C,D).

From the coefficients of the main handshake subphase model (Figure 3B), the following can be stated. Since the handshake experiment was done approximately at a similar height, the low variability of the intercept (*A*_*V*3_) can be expected. The low variability of the angular frequency (*ω*) obtained in the conducted experiment indicates that the handshake is mostly performed at an angular frequency of 17.7 (SD = 2.4) rad s^−1^. The high variability of *A*_*V*2_ of 16.2 (SD = 2.1) and *γ* of 1.8 (SD = 1.2) signify that the features they represented (downward trend and exponential decay, respectively) were not norms. In this work, their mean values were used in the handshake model. However, one may want to use smaller or larger values and recalculate the affected phases and subphases to arrive to a different vertical hand motion model. This can be investigated further to impart personalities for robot handshakes.

### 6.3 Comparisons: Model vs. Experiments

The proposed hand movement models agreed well with the experimental datasets (Figures 4A–F). The general trend of the handshake positions in the *y* and *x*-axes were achieved by the model (Figures 4A,D), but there were marked differences in the displacement values. The velocities and accelerations in the *y* and *x*-axes were likewise represented.

However, there are some differences that are worth noting. First, the handshake duration of the model was slightly less than that of the dataset by 0.25 s (Figures 4A,B), yet the model was able to capture the amplitude in the experimental data. Second, the model has a higher exponential damping and downward trend, which resulted in less velocity and acceleration values toward the end of the main handshake subphase. Third, even after filtering the data, the acceleration plots show some fluctuations at the reaching and return phases that prevented the observation of the trends at those regions (Figures 4B,C,E,F). This is because the experimental results were position data, which were numerically differentiated. Due to the subtractive nature of differentiation, the errors were amplified (Chapra, 2012). For one to see the velocity and acceleration trends, a better approach is to use a sensor type that matches the variable of interest.

Taken together, the vertical displacement had a mean difference of 3.3 cm (SD = 1.25 cm) in the peaks of the handshake oscillations and a mean difference of 2.3 cm (SD = 0.48 cm) in the throughs (Figure 4A). The horizontal displacement had a mean difference of 6.35 cm (SD = 0.86 cm) from the initial reach to the release of contact (Figure 4D). These differences between the model and experimental results might be tolerable variances in an actual handshake when the heights of the handshaking partners and their relative proximity to one another are considered. The velocities and accelerations in both the *y* and *x*-axes will be more perceivable during actual handshake, which our model was able to capture well.

### 6.4 Comparisons With Other Human-Robot Handshakes

**FIGURE 5:**
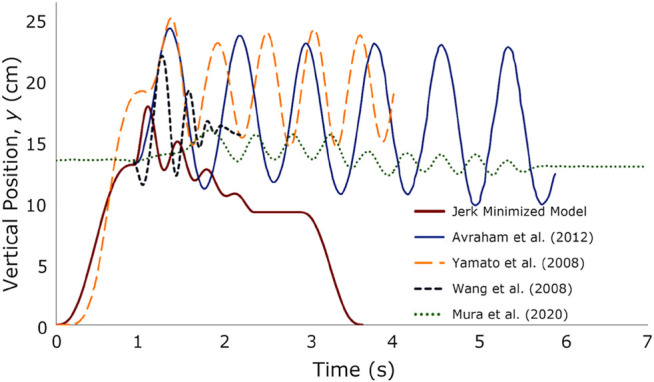
Vertical hand movement comparisons of human-to-human handshake against human-robot handshake [data extracted from Avraham et al. (2012); Yamato et al. (2008); Wang et al. (2008); Mura et al. (2020)].

To determine whether humanoid robots are now able to perform handshakes that are similar to humans, we plotted the vertical position plots from the jerk-minimized handshake model and the previously published results (Figure 5). The differences between the proposed model and the other models could be due to cultural differences and social norms. There were evidences that have already reported ethnic as well as gender differences in handshakes (Chaplin et al., 2000; Katsumi et al., 2017; Melnyk and Henaff, 2019).

The differences could also be due to the following reasons. First, the amplitude of the shaking motion is too large such that it is comparable in magnitude to the displacement of the hand in the reaching phase (e.g., Figure 5; 14.7 cm in Avraham et al. (2012) vs. 13 cm *y*-axis displacement during reaching). Second, the number of oscillations were more than 5 in Yamato et al. (2008), Avraham et al. (2012), Mura et al. (2020), while our model had 4 oscillations. Wang et al. (2008) also showed 4 oscillations. Moreover, the duration of the other handshakes exceeded 4 s while our model had less than 4 s duration for a full handshake. In Wang et al. (2008), it can be observed that the duration of the contact phase was similar to our model. In Table 11, our model had a contact time of 1.95 s while a shorter contact duration of 1.25 s was observed in Wang et al. (2008). Others had more than 3 s of contact time.

**TABLE 11:** Comparisons between the Handshake phase Durations of the multiphase Jerk-Minimized Model and Earlier Models (in sec).

Model name	Reaching time	Contact time*	No motion during contact	Return time	Total time
Multiphase Jerk-Minimized Model	0.92	1.95	0.52	0.75	3.63
Wang et al. (2008)	—	∼1.25	—	—	1.25
Yamato et al. (2008)	∼1.09	∼2.90	—	—	3.99
Avraham et al. (2012)	—	∼4.98	—	—	4.98
Mura et al. (2020)	∼0.74	∼4.92	—	∼1.32	6.98

* Excluding no motion during contact.

The percentage difference between the contact phase of our model and that in Yamato et al. (2008), Wang et al. (2008), Mura et al. (2020), and Avraham et al. (2012) are 39.18, 43.75, 86.46, and 87.45%, respectively. Additionally, our model, as well as that in Wang et al. (2008), exhibited exponential decay in the amplitude in the contact phase. The oscillations in Mura et al. (2020) slightly displayed this behavior while the handshake in Yamato et al. (2008) and Avraham et al. (2012) did not show this decay.

Third, other models only considered the contact phase of the handshake and not the complete reaching-contact-return phases. The handshake movement presented in Avraham et al. (2012), which was plotted such that its starting point matches the start of the contact phase, was only limited to the handshaking motion. In contrast, the model of Yamato et al. (2008) included the handshake contact movement up to the end of shaking. Post-contact handshake phases were not shown in the other models, except in our model and that in Mura et al. (2020). However, the horizontal lines in their reaching and post-contact phases were near the same level as the baseline heights of the robot arm when the participant and the robot started and completed the handshake. In Figure 5 and in Table 11, our model showed the subphase of no motion of 0.52 s just before both hands disengaged in the return phase.

## 7 Discussion

We will discuss the sources of variability in human-human handshakes and provide insights for lifelike robot handshake. We will elaborate on the guidelines for natural pHRI handshakes.

### 7.1 Variability in Human-Human Handshakes

The mean handshake duration in our experiments was 3.63 s (SD = 0.45 s) (Table 3). Previous works confirm the variability in the handshake duration (Prasad et al., 2021). Others had a minimum of 0.73 s (SD = 0.08 s) to a maximum of 4.05 s (SD = 0.53 s) duration of handshakes (Melnyk et al., 2014; Tagne et al., 2016; Melnyk and Henaff, 2019). The duration of the contact phase was observed to vary considerably when compared to the other handshake phases (Table 3). The standard deviation (SD) for the reaching and return phases were relatively small at 0.1 and 0.12 s, respectively. The SD for the contact phase was 0.46 s. The reason behind this is the variability of the handshake characteristics of each participant. For example, participants who prefered to perform two or three oscillations would generally be going to shake hands for a shorter duration than the participants who perform more oscillations.

In human handshakes, it appears that the hand undergoes planned and unplanned movements once contact has been initiated. The unplanned movement is the movement executed as the initiator and receiver correct their hand postures. This movement depends on how contact is first made. The first contact location would be the web of the hands. The accepted practice is to keep an eye to eye contact with the other person before, during, and after the handshake. In object grasping, humans gaze briefly to the affordances of an object before grasping it (Johansson et al., 2001; Juravle et al., 2015).

In handshakes, one has to estimate the location of the other person’s hand (de la Rosa et al., 2014; Fademrecht et al., 2016). If the targets were not reached correctly, perhaps due to miscalculation from peripheral vision, the handshake partners will try to correct their hand positions while proceeding with the handshake phases. This results in some randomness in the initial location that comes into contact. While the objective of this paper was to model the planned movement, it is also important to account for the unplanned movements in robot handshake algorithms with the correct estimation of the location of the handshake partner’s hands during the reaching phase.

### 7.2 Toward Lifelike Human-Robot Handshakes

While there are some variabilities in human-to-human handshakes, there are elements which make a handshake acceptable. Some of these were described in this work: the grasping forces, minimal movements in the *z*-plane, the amplitude of the handshake, the phases of movement, and the duration of each phase. Other parameters that are beyond the scope of the current paper are: the eye contact between the handshaking partners, the fingers’ micro-movements during handshake, the softness and warmth of the hands. A significant deviation in one of these parameters will be judged as an odd or unnatural handshake, which will lead to an awkward interaction (Kadambi et al., 2020).

The comparisons in Figure 5 led us to some insights toward lifelike human-robot handshakes. Across the different models, our model showed all the phases of the handshake movement from the reaching to the release. The contact phase has the subphases of preshake, handshake, postshake, and no motion. Others have only investigated the contact phase. In one model, there were up to 9 handshake oscillations. In another, the vertical hand movements showed amplitudes of 12 cm that were monotonous and do not show a decay in the vertical position. If those handshake movements were done during a human-to-human or robot-to-human interaction, these will be perceived as unnatural. We also showed a period of no motion of around half a second just before the release of contact between the handshake. Due to the multiphase modeling of the handshake phases and the subphases that we implemented, we were able to discover this no-motion behavior. This non-movement may have implications on promoting better social interactions. We will have to further test if there is such an effect in a future study.

As humanoid robots begin to collaborate and co-exist with humans (Khatib et al., 1999; Heinzmann and Zelinsky, 2003; Haddadin et al., 2009), it is conceivable that the human interaction partner will expect handshakes. Just like in human interactions, it is probable that humans will make split-second judgements on whether a humanoid robot possesses humanlike traits through a handshake (e.g., Is this robot sociable or competent enough to interact with me?). Thus, a humanoid robot should be able to know what to do when a person extends his or her hand (Cabibihan et al., 2009; Knoop et al., 2017; Avelino et al., 2018).

The validation of a realistic humanoid robotic handshake requires a human-robot experiment where a handshaking robot follows the proposed trajectory that we showed and the human’s perception of the handshake will be recorded. Our future work would be a follow-up experiment involving studies on human-robot handshake interaction, in which we have to consider what is the desired social relationship a human or robot would like so that the appropriate type of handshake can be used during an interaction (e.g., friendly, professional, acquaintance, etc.,) by the robot.

### 7.3 Guidelines for pHRI Handshakes

With the multiphase model revealing some of the nuances of human to human handshakes, we provide guidelines for lifelike handshakes during physical human-robot interactions.

#### 7.3.1 Guideline 1: Improve Emotional Connection With a “No Motion” Subphase Before Grasp Release

There are three phases in a handshake: reaching, contact, and return. A full handshake takes around 3.63 s. The reaching and return phases take 0.92 and 0.75 s, respectively. The contact phase, where the up and down handshake motion occurs, takes around 1.96 s. The contact phase can be further divided into subphases for the vertical motion (*y*-axis plane) and horizontal motion (*x*-axis plane). The vertical motion has the following subphases: preshake (0.06 s), main handshake (1.31 s), postshake (0.06 s), and a period of no motion (0.52 s). The horizontal motion has the subphases of: motion (0.91 s) and no motion (1.44 s). The period of no motion was a novel finding that we showed in this work. A humanoid robot can use that time for other verbal or non-verbal forms of communication. For example, it can be used for greeting and saying the person’s name. That additional 0.5 s of hand-holding can also communicate softness and warmth to the human handshaking partner through soft and warm artificial skin. In the classical experiments of Harlow and Zimmermann (1958) on affection, they identified softness, warmth, and comfort to be among the basic physiological need of humans. Soft and warm artificial skins have been developed that can be incorporated into compliant robotic hands for handshaking [e.g., Cabibihan et al. (2006b); Cabibihan et al. (2015); Knoop et al. (2017)].

#### 7.3.2 Guideline 2: Use Prediction Schemes For Trajectory Estimation

Earlier works by Wang et al. (2008) and Avraham et al. (2012) did not consider the reach and return phases of the handshake as these works only focused on the contact phase. In the present work, we found the duration of reaching, contact, and return phases corresponded to 25, 54, and 21%, respectively, of the whole handshake movement. Moreover, the vertical movement for reaching was faster than the return. The rate of reaching the other person’s hand was 14.16 cm s^−1^ while the disengagement of contact and return of the hand to the neutral position was -9.78 cm s^−1^. Should a multiphase handshake model be implemented in a robot to shake hands with a human, these velocities are important so as not to keep the human partner waiting if the reach is too slow or to hurt the human if the reach is too abrupt.

The robot would need to be equipped with a vision system and a well-coordinated fingers-hand-arm system that can estimate the location of the handshaking partner’s hand in around 0.9 s with a velocity of around 14 cm s^−1^. The humanoid should be able to make predictions on where the web of the handshaking partner’s hand will land, in consideration of partner’s body height and width (i.e., to estimate the sagittal plane or the plane that divides the body into the left and right parts). In Saxena et al. (2008), they presented a probabilistic model that estimated the possible grasping points of novel objects in 3D space. With at least two images of an object, their algorithm identified a few points in the image that corresponded to the target locations for grasping. This set of points was triangulated to obtain the 3D location for grasping. In Park et al. (2019), they described an intention-aware motion planning algorithm that was able to compute smooth trajectories based on the predicted actions of a human.

#### 7.3.3 Guideline 3: Compensate For the Initial Position Uncertanties With Admittance Control Schemes

With reference to Figure 2, the *x*-position (blue) shows that overshoots occurred before a brief steady state behavior. The overshoots in the vicinity of start of the contact phase can be interpreted as the over-reaching of the elbow and wrist movement toward the partner. For the *z*-position (green), there was a movement towards the sagittal body plane of the hand shaking partner. While both *x* and *z* positions were stabilizing, the oscillations on the *y*-position (red) commenced execution. It can be expected that there will be variability on the overshoots depending on the distance and angle when the handshake starts.

Because the human partner can have an infinite combination of handshake approaches, the robot should be equipped with high admittance (or low impedance) control system (Keemink et al. (2018) for design guidelines on admittance control for pHRI). The admittance or impedance controller will execute when contact occurs (i.e., during the preshake, handshake, postshake, and no motion subphases). For a robotic implementation, the admittance or impedance controller will have to rely on the desired social closeness between the two handshaking partners on whether one gives a vigorous, neutral, or perhaps a limp handshake. The actual admittance control parameters needed for those handshake styles will require additional study. There can be factors like social relationship and emotions that one would like to convey to another (e.g., excitement, condolences, agreement, neutral). One or a combination of these factors will influence the admittance parameters at the contact phase.

One can then have an admittance controller to follow the desired handshake trajectory. As the trajectory is being followed and a disturbance on the arm occurs, the controller will determine the stiffness of the robot arm. To recall, there are three dynamics-based parameters that are involved: mass properties, damping, and the stiffness. Following the trajectory, the stiffness will have to be specified to the admittance controller. In a potential future scenario when a human partner meets a humanoid robot partner, they might exchange a “firm handshake”. A firm handshake corresponds to high stiffness achieved by the controller. In a future study, the mass property, damping, and stiffness will be determined. We can use known dynamics-based methods to follow the desired trajectory under impedance control in consideration of those three parameters [Hogan (1985b,a); Keemink et al. (2018)]. In other words, if the dynamics of the robot is known and the required dynamics is also known, one can then use known methods to control the system to achieve the desired dynamic behavior. For a realistic handshake, the controller has to follow the trajectories that we proposed together with the required admittance or impedance, which is based on the appropriate social behavior of the robot.

#### 7.3.4 Guideline 4: Implement a Gradual Decay in Handshake Amplitudes to Avoid Monotony

Depending on the handshake partner, there can be 1 to 5 handshake oscillations (or even more). Our model showed that human handshakes oscillate in a sinusoidal manner and exhibit an exponential decay. The handshake amplitude was calculated to be 5.3 cm at the start and 0.2 cm at the end. In addition to this, both hands went on upward trajectories just after the contact commenced and the peak vertical positions were reached (e.g., Figures 2A, 4A). A humanoid robot arm would need to synchronize with the human handshaking partner’s movements. Many of the robot handshake algorithms have already implemented some type of leader-follower synchronization algorithms [e.g., Avraham et al. (2012); Mura et al. (2020)]. Similar algorithms could benefit from implementing a gradual decay in the handshake amplitude to mimic human-human handshakes and to avoid monotonous movements [e.g., Schillaci and Hafner (2011); Kwiatkowski and Lipson (2019) on randomness in lifelike robot movements]. Moreover, human-human handshakes first go upwards upon contact. In Figure 5, the human-robot handshake examples followed that trend except in the handshake in Wang et al. (2008), where the handshake went 2 cm downward before going upward to reach the peak vertical handshake position.

#### 7.3.5 Guideline 5: Calibrate the Robot Hand For Safe Handshake Grasping

The appropriate contact forces will have to be absorbed or transmitted to create a realistic feel of a handshake. In the work of Avraham et al. (2012), for example, they used a stylus as a representation of the hand (Phantom Desktop haptic device, SensAble Technologies, United States). In the current work, the recorded grasping forces were around 2 N on 6 locations of the hand at the proximal and middle phalanges of little finger, at middle phalanges of the ring and middle fingers, at the distal phalange of the thumb, and the back of the palm (Figure 1). The largest forces of around 8 N were recorded at the hypothenar eminence (i.e., fleshy part at the base of the palm). Such distribution of forces is not possible with a stylus (Avraham et al., 2012) or with an artificial handshaking hand that are not articulated (Kasuga and Hashimoto, 2005; Yamato et al., 2008) or having stiff fingers and planar palms (Mura et al., 2020).

For an implementation with a robotic hand, these imply that the human partner’s hand will have to be fully enveloped. During handshake, the robot’s fingers will press the handshake partner’s hand mostly toward the fleshy part of the palm. The contact sensor data we collected showed that normal contact forces from the fingers was opposed by contact sensors on the palm. The force balance equation showed that 98.61% of the contact forces was accounted for in the sensor locations in Figure 1B (cf. Section 4; 1.39% percent difference). The robot hand needs 4 or 5 fingers to fully and safely grasp the other person’s hand. There have been several compliant hands that have already been developed [e.g., Aukes et al. (2014); Subramaniam et al. (2020); Hussain et al. (2020)]. Designs for safe grasping have also been incorporated into the compliant hands through soft artificial skins and joints that permit over exertion [e.g., Catalano et al. (2014); Alkhatib et al. (2019); Mura et al. (2020)]. To achieve safe grasps with the human partner’s hands, compliant robot hands should be able to exert grasping forces of up to 10 N. Soft, compliant artificial skin on the location equivalent to the hypothenar eminence of the robot’s palm would also help as the robot’s fingers will press the human hand toward this location.

## 8 Conclusions and Future Work

Humans are social by nature. They constantly communicate with one another through verbal and non-verbal forms of communication. Throughout human history, a simple gesture like the handshake has contributed toward improving human relationships in negotiations, employment, in communicating empathy, and in diffusing conflicts (Stewart et al., 2008; Katsumi et al., 2017; Schroeder et al., 2019).

For humanoid robots to convey trust and competence in physical Human-Robot Interaction (pHRI), we developed guidelines that captured how we can mimic human handshakes for humanoid robots. This paper investigated the fine nuances of human-to-human handshake. We developed a multiphase jerk minimization model with exponential decay to replicate the smooth human-human handshake motion. We then evaluated whether the prevalent human-robot handshake models were comparable to our model. By comparing the available handshake models, we proposed 5 guidelines that could help achieve a more natural human-robot handshake. These guidelines are:

1) Improve emotional connection with a “no motion” subphase before grasp release;
2) Use prediction schemes for trajectory estimation;
3) Compensate for the initial position uncertanties with admittance control schemes;
4) Implement a gradual decay in handshake amplitudes to avoid monotony;
5) Calibrate the robot hand for safe handshake grasping.

The oscillatory model proposed for the handshaking phase has a mathematical limitation that warrants the contact phase to be divided into subphases. The limitation comes from the fact that the derivatives of a sinusoidal function cannot be zero at the same time. With this in mind, one cannot extend the sinusoidal model in either direction hoping to obtain a point where the velocity and acceleration are both zero (to satisfy the static equilibrium state conditions). However, it is clear that the handshaking motion is sinusoidal. This led to the proposal of the transitional subphases in the preshake and postshake.

Another limitation of this work was the small sample size of the participants which was made necessary due to the large number of sensor data that was gathered. However, the data from the sensors agree with the earlier work in Cabibihan et al. (2011) where the handshake grasping forces of 2 N and above were reported. Additionally, Nagy et al. (2020) reported that the duration of a normal handshake was around 3 s. They further reported that a prolonged handshake (beyond 5 s) can create an emotional discomfort. The participant sizes in Cabibihan et al. (2011) and in Nagy et al. (2020) were 30 and 34, respectively.

It is also acknowledged that there are cultural and gender differences in handshakes (Bernieri and Petty, 2011; Katsumi et al., 2017; Smith, 2020). The study could be improved by increasing the number of participants in the roles of handshake initiators and receivers from different cultures. In addition, experiments that consider the other gender pairings (i.e., female-female, female-male) can be investigated. Future work could implement the handshake movements described in this work in a robot handshake platform and verify whether the handshake is humanlike or not, in terms of arm and hand motions and the emotions that the handshake evoke. It is probable that humanoid robots in the future will be given a gender. Thus, it would be worthwhile to investigate handshakes that can convey a humanoid robot’s personality (e.g., aggressive, modest, professional).

The handshake has withstood the test of time amid wars and pandemics. Handshakes will continue to be an important gesture of greeting and goodwill. Looking forward, there is an expectation that robots will get more accepted in human society. The results of this work and the guidelines that we proposed may help accelerate the development of robots that will routinely perform more natural handshakes as they interact with their human partners.

## Data Availability

The raw data supporting the conclusion of this article will be made available by the authors, without undue reservation.
