# Supplementary material for: Guidelines for Robot-to-Human Handshake From the Movement Nuances in Human-to-Human Handshake
Source: Front Robot AI. 2022 Mar 28;9:758519. doi: 10.3389/frobt.2022.758519 (PMC8996188; doi:10.3389/frobt.2022.758519)
Supplement: Supplementary file 1 [file DataSheet1.zip › Supplementary/osa-supplemental-document-template.pdf]

Guidelines for Robot-to-Human Handshake From the Movement Nuances in Human-to-Human Handshake (Supplementary Material)

The steps in determining the polynomial equations are included here under the conditions sets encountered in this work. The procedures were generalized to allow the derived equations to be used under conditions other than the ones encountered in this work (e.g. between two dynamic equilibrium states).

1. APPENDIX A: DETERMINATION OF THE POLYNOMIAL EQUATION UNDER FIVE CONDITIONS (UNKNOWN START POSITION)

Under the following conditions where the start position y_0 is unknown, and D is the duration,

$$\begin{aligned} &y(0) = y_0 \rightarrow \text{unknown} \quad && y'(0) = y'_0 \quad && y''(0) = y''_0 \\ &y(D) = y_1 && y'(D) = y'_1 && y''(D) = y''_1 \end{aligned}$$

The polynomial has the form of Eqn Eq. (A1). The steps to determine the coefficients are shown below.

$$y(t) = C_4 t^4 + C_3 t^3 + C_2 t^2 + C_1 t + C_0 \tag{A1}$$

Differentiating Eqn. Eq. (A1) twice, the following equations are obtained:

$$y'(t) = 4C_4 t^3 + 3C_3 t^2 + 2C_2 t + C_1 \tag{A2}$$

$$y''(t) = 12C_4 t^2 + 6C_3 t + 2C_2 \tag{A3}$$

Substituting $t = 0$ in Eqns. Eq. (A2) and Eq. (A3), and simplifying,

$$C_1 = y'_0 \tag{A4}$$

$$C_2 = \frac{y''_0}{2} \tag{A5}$$

Substituting $t = D$, C_1, and C_2 in Eqn. Eq. (A2) and Eq. (A3), and simplifying,

$$y'_1 = 4C_4 D^3 + 3C_3 D^2 + y''_0 D + y'_0 \tag{A6}$$

$$y''_1 = 12C_4 D^2 + 6C_3 D + y''_0 \tag{A7}$$

Rearranging and writing in matrix notation,

$$\begin{bmatrix} y'_1 - y''_0 D - y'_0 \\ y''_1 - y''_0 \end{bmatrix} = \begin{bmatrix} 4D^3 & 3D^2 \\ 12D^2 & 6D \end{bmatrix} \begin{bmatrix} C_4 \\ C_3 \end{bmatrix}$$

Solving for C_3 and C_4,

$$C_3 = \frac{y'_1 - y'_0}{D^2} - \frac{2y''_0 + y''_1}{3D} \tag{A8}$$

$$C_4 = \frac{y'_0 - y'_1}{2D^3} + \frac{y''_0 + y''_1}{4D^2} \tag{A9}$$

Substituting $t = D$, C_1, C_2, C_3, and C_4 in Eqn. Eq. (A1), solving for C_0, and simplifying,

$$C_0 = \frac{y''_1 - y''_0}{12} D^2 - \frac{y'_0 + y'_1}{2} D + y_1 \tag{A10}$$

To obtain y_0, $t = 0$ is substituted in Eqn. Eq. (A1),

$$y_0 = C_0 = \frac{y''_1 - y''_0}{12} D^2 - \frac{y'_0 + y'_1}{2} D + y_1 \tag{A11}$$

2. APPENDIX B: DETERMINATION OF THE POLYNOMIAL EQUATION UNDER FIVE CONDITIONS (UNKNOWN END POSITION)

Under the following conditions where the end position y_1 is unknown, and D is the duration,

$$\begin{aligned} y(0) &= y_0 & y'(0) &= y'_0 & y''(0) &= y''_0 \\ y(D) &= y_1 \rightarrow \text{unknown} & y'(D) &= y'_1 & y''(D) &= y''_1 \end{aligned}$$

The polynomial has the form of Eqn. Eq. (A1), and the steps to determine the coefficients are the same as the ones shown in Appendix A except for determining C_0 and for calculating y_1.

To obtain C_0, $t = 0$ is substituted in Eqn. Eq. (A1) and the result is simplified to get:

$$C_0 = y_0 \tag{B1}$$

To calculate y_1, $t = D$, C_0, C_1, C_2, C_3, and C_4 are substituted in Eqn. Eq. (A1) and the result is simplified to get:

$$y_1 = \frac{y''_0 - y''_1}{12} D^2 + \frac{y'_0 + y'_1}{2} D + y_0 \tag{B2}$$

3. APPENDIX C: DETERMINATION OF THE POLYNOMIAL EQUATION UNDER SIX CONDITIONS

Under the following conditions where D is the duration,

$$\begin{aligned} y(0) &= y_0 & y'(0) &= y'_0 & y''(0) &= y''_0 \\ y(D) &= y_1 & y'(D) &= y'_1 & y''(D) &= y''_1 \end{aligned}$$

The polynomial has the form of Eqn. Eq. (C1). The steps to determine the coefficients are shown below:

$$y(t) = C_5 t^5 + C_4 t^4 + C_3 t^3 + C_2 t^2 + C_1 t + C_0 \tag{C1}$$

Differentiating Equation Eq. (C1) twice, the following equations were obtained:

$$y'(t) = 5C_5 t^4 + 4C_4 t^3 + 3C_3 t^2 + 2C_2 t + C_1 \tag{C2}$$

$$y''(t) = 20C_5 t^3 + 12C_4 t^2 + 6C_3 t + 2C_2 \tag{C3}$$

Substituting $t = 0$ in Eqns. Eq. (C1), Eq. (C2) and Eq. (C3), and simplifying,

$$C_0 = y_0 \tag{C4}$$

$$C_1 = y'_0 \tag{C5}$$

$$C_2 = \frac{y''_0}{2} \tag{C6}$$

Substituting $t = D$, C_0, C_1, and C_2 in Eqn. Eq. (C1), Eq. (C2) and Eq. (C3), and simplifying,

$$y_1 = C_5 D^5 + C_4 D^4 + C_3 D^3 + (y''_0)/2D^2 + y'_0 D + y_0 \tag{C7}$$

$$y'_1 = 5C_5 D^4 + 4C_4 D^3 + 3C_3 D^2 + y''_0 D + y'_0 \tag{C8}$$

$$y''_1 = 20C_5 D^3 + 12C_4 D^2 + 6C_3 D + y''_0 \tag{C9}$$

Rearranging and writing in matrix notation,

$$\begin{bmatrix} y_1 - \frac{y''_0}{2} D^2 - y'_0 D - y_0 \\ y'_1 - y''_0 D - y'_0 \\ y''_1 - y''_0 \end{bmatrix} = \begin{bmatrix} D^5 & D^4 & D^3 \\ 5D^4 & 4D^3 & 3D^2 \\ 20D^3 & 12D^2 & 6D \end{bmatrix} \begin{bmatrix} C_5 \\ C_4 \\ C_3 \end{bmatrix}$$

Solving for C_3, C_4, and C_5,

$$C_3 = \frac{y_1'' - 3y_0''}{2D} - \frac{2(3y_0' + 2y_1')}{D^2} + \frac{10(y_1 - y_0)}{D^3} \tag{C10}$$

$$C_4 = \frac{\frac{3}{2}y_0'' - y_1''}{D^2} + \frac{8y_0' + 7y_1'}{D^3} + \frac{15(y_0 - y_1)}{D^4} \tag{C11}$$

$$C_5 = \frac{y_1'' - y_0''}{2D^3} - \frac{3(y_0' + y_1')}{D^4} + \frac{6(y_1 - y_0)}{D^5} \tag{C12}$$
